# Editorial: Food and nutrition for athletics: redefining the role and application

**DOI:** 10.3389/fnut.2025.1602040

**Published:** 2025-04-14

**Authors:** Baoming Tian, Xiao Li Shen

**Affiliations:** ^1^College of Food Science and Technology, Zhejiang University of Technology, Huzhou, China; ^2^Key Laboratory of Food Macromolecular Resources Processing Technology Research (Zhejiang University of Technology), China National Light Industry, Hangzhou, China; ^3^School of Public Health, Zunyi Medical University, Zunyi, Guizhou, China; ^4^Key Laboratory of Maternal and Child Health and Exposure Science, Guizhou Provincial Department of Education, Zunyi, Guizhou, China

**Keywords:** athletic performance, macronutrients, carbohydrate, protein, bioactive plant compounds

Food and nutrition play a vital role in athletes' health and performance, making it a timeless topic. Of course, this also applies to the general population. This Research Topic focuses on two main aspects: the impact of macronutrients on athletic performance and the effects of bioactive plant compounds on exercise performance.

Bagheri et al. reported that variations in muscle strength, power adaptation, and endurance after 16 weeks of either concurrent or resistance training with varying high-protein intakes were not linked to changes in lean mass among resistance-trained young males. Through meta-analysis, Zhao et al. concluded that protein intake offers modest benefits to athletes, especially in improving endurance. Subgroup analysis indicates that protein intake boosts muscle glycogen levels and that combining protein with carbohydrates is more effective for endurance athletes than consuming high protein alone. Kuhlman et al. reported that male collegiate gymnasts may have a high prevalence of low energy availability, primarily due to insufficient relative energy and carbohydrate intake. Ritson et al. observed a drug-free bodybuilder following evidence-based nutrition strategies over 18 weeks of low energy availability, resistance training, and a high-protein diet to attain extreme leanness, providing insights into the fluctuations of free triiodothyronine and total testosterone. Kripp et al. reported that in active and healthy individuals, a low-carbohydrate, high-fat diet negatively impacts individual blood lipid profiles compared to carbohydrate-rich diets. Noakes and Prins analyzed and highlighted the potential limitations of the exogenous carbohydrate ingestion prediction model for achieving a sub-2 h marathon, as proposed by Lukasiewicz et al. (1). Zhang et al. discovered that in free-living conditions, athletes' body composition is influenced by habitual water intake rather than hydration status.

Ikeda et al. found that kaempferol, a flavonoid found in edible plants, enhances sleep quality and may contribute to long-term improvements in quality of life, including physical activity, as demonstrated in a double-blind, placebo-controlled, crossover trial. Guo and Rezaei reviewed the effects of ashwagandha (*Withania somnifera*), an herbal plant from the Solanaceae family, highlighting its ability to enhance antioxidant response, alleviate stress-related conditions such as depression, anxiety, and insomnia, and improve physical performance in sports such as maximum oxygen consumption, treadmill time to exhaustion, metabolic equivalents, and more. Wang et al. explored strategies to enhance the bioavailability of *Rhodiola rosea*, finding that its nano-dosage form significantly improves anti-exercise fatigue effects in rats, particularly when combined with aerobic exercise, compared to the normal form.

The Research Topic covered in this issue are limited. In the future, researchers can conduct in-depth studies on various areas such as the specific nutritional requirements of different types of sports (2), the effects and mechanisms of precise formulations on specific athletic performance (3), the potential toxicity and underlying mechanisms of long-term supplementation of certain nutrients (4), and the impact and mechanisms of specific nutrients on injury prevention or rehabilitation in athletes.
